# Outcomes of lobectomy on pulmonary function for early stage non‐small cell lung cancer (NSCLC) patients with chronic obstructive pulmonary disease (COPD)

**DOI:** 10.1111/1759-7714.13445

**Published:** 2020-05-06

**Authors:** Sen Wei, Feng Chen, Renwang Liu, Dianxun Fu, Yanye Wang, Bo Zhang, Dian Ren, Fan Ren, Zuoqing Song, Jun Chen, Song Xu

**Affiliations:** ^1^ Department of Lung Cancer Surgery Tianjin Medical University General Hospital Tianjin China; ^2^ Tianjin Key Laboratory of Lung Cancer Metastasis and Tumor Microenvironment, Lung Cancer Institute Tianjin Medical University General Hospital Tianjin China; ^3^ Department of Radiology Tianjin Medical University General Hospital Tianjin China

**Keywords:** COPD, lobectomy, non‐small cell lung cancer, pulmonary function

## Abstract

**Background:**

Lung cancer is the first cause of cancer mortality worldwide. Chronic obstructive pulmonary disease (COPD) is an independent risk factor for lung cancer. An epidemiological survey discovered that the presence of COPD increases the risk of lung cancer by 4.5‐fold. Lobectomy is considered to be the standard surgical method for early stage non‐small cell lung cancer (NSCLC). However, the influence of lobectomy on the loss of pulmonary function has not been fully investigated in NSCLC patients with COPD.

**Methods:**

We searched the PubMed database using the following strategies: COPD and pulmonary function test (MeSH term) and lobectomy (MeSH term) from 01 January 1990 to 01 January 2019. We selected the articles of patients with COPD. A total of six studies, including 195 patients with COPD, provided lung function values before and after surgery.

**Results:**

Five out of six studies focused on the short‐term change of pulmonary function (within 3–6 months) after lobectomy, and the average loss of FEV1 was 0.11 L (range: −0.33–0.09 L). One study investigated the long‐term change of pulmonary function (within 1–2 years) after lobectomy, and the average loss of FEV1 was 0.15 L (range: −0.29–0.05 L).

**Conclusions:**

A short‐term (3–6 months) loss of pulmonary function after operation is acceptable for lung cancer patients with COPD. However, there may be a high risk of postoperative complications in NSCLC patients with COPD. Therefore, surgical treatment needs to be carefully considered for these patients.

## Introduction

Lung cancer is the first cause of cancer mortality worldwide. Almost one third of patients with non‐small cell lung cancer suffer (NSCLC) suffer from other lung diseases, such as chronic obstructive pulmonary disease (COPD) and emphysema,^1^ and exhibit a much worse preoperative pulmonary function. In recent years, studies have shown that COPD is an independent risk factor for lung cancer irrespective of smoking status.^2^ The underlying relationship between COPD and lung cancer has been widely studied, including genetic susceptibility, DNA damage and repair, epigenetics, downregulation of specific microRNA, expression of proinflammatory genes, and adaptive immune response.^3^

Lobectomy is considered to be a standard surgical method for early stage NSCLC because of the high local recurrence rates after sublobar resection (SR). A reduction in averaged forced expiratory volume in one second (FEV1) values is associated with increased risk of lung cancer and mortality.^4^ If the preoperative FEV1 of patients undergoing lobectomy is greater than 1.5 L, the mortality rate can be less than 5%. When the preoperative FEV1 of patients with COPD is less than 1.5 L, sublobar resection may be considered.^5^ Therefore, in clinical practice, clinicians are more likely to refuse lobectomy and select a more limited sublobar resection based on lower FEV1 prior to surgery. However, there is lack of robust evidence of how surgery, especially lobectomy, influences the postoperative loss of pulmonary function and treatment‐related complications.

In this article, we reviewed and analyzed all the publications in early‐stage NSCLC patients with COPD disease who underwent lobectomy in order to compare the change in pulmonary function before and after surgery.

## Methods

We searched the PubMed database using the following search terminology: COPD and pulmonary function test (MeSH term) and lobectomy (MeSH term) and lung cancer or pulmonary cancer (MeSh term). Only English articles and human studies published from 01 January 1990 to 01 January 2019 were included. All articles were manually identified to meet the requirements of patients with COPD (FEV1/FVC < 70% of predicted value). All the included studies also provided exact pulmonary function value or the difference before and after surgery (Table 1).

**Table 1:** Clinical characteristics of patients in the selected experimental group

						Gender		Smoking		
First author	Year	Surgical approach	Number	Age	Period	Female	Male	Yes	No	Note
Bobbio A^6^	2005	Open	11	65 ± 8	2003–2004	9	3	NA	NA	
Korst RJ^7^	1998	Open	19	63.5	1995–1996	9	10	NA	NA	FEV1 ≤ 60% of predicted
	1998	Open	13	62.8	1995–1996	6	7	NA	NA	FEV1>60% of predicted
Kushibe K^8^	2009	Unknown	16	66.3 ± 6.6	2004–2007	0	16	16	0	RUL
	2009	Unknown	15	69 ± 3.9	2004–2007	1	14	13	2	LUL
	2009	Unknown	11	69.8 ± 9.8	2004–2007	0	11	11	0	RLL
	2009	Unknown	11	71.2 ± 4.1	2004–2007	3	8	9	2	LLL
Sekine Y^9^	2003	Open	48	66 ± 6.6	1990–2000	2	46	46	2	
Schattenberg^10^	2007	Unknown	16	70	2000–2006	NA	NA	NA	NA	
Subotic DR^11^	2007	Unknown	35	57 (45–72)	NA	7	28	NA	NA	

FEV1, forced expiratory volume; RUL, right upper lobe; RLL, right left lobe; LUL, left upper lobe.

**Table 2:** Change in pulmonary function before and after lobectomy

			Baseline value				Postoperative value			
	First author	Number	FVC	FVC%	FEV1	FEV1%	FVC	FVC%	FEV1	FEV1%
Short‐ term	Bobbio A^6^	11	NA	NA	1.4 ± 0.5	53 ± 20	NA	NA	1.4 ± 0.5	53 ± 18
	Kushibe K^8^	16	2.97 ± 0.63	91.3 ± 19.2	1.64 ± 0.67	66.5 ± 24.7	NA	87	1.73	70.2
	Kushibe K^8^	15	2.72 ± 0.64	85.3 ± 17.1	1.59 ± 0.53	69.7 ± 21.4	NA	76.3	1.55	67.8
	Kushibe K^8^	11	2.8 ± 0.52	87.8 ± 11.8	1.8 ± 0.36	79 ± 15	NA	69.3	1.47	64.6
	Kushibe K^8^	11	2.77 ± 0.56	92.7 ± 11.1	1.69 ± 0.45	82.7 ± 17.5	NA	79.9	1.53	74.8
	Schattenberg^10^	16	2.4	84	1.3	60	2.1	79	1.2	57
	Subotic DR^11^	35	NA	NA	1.59	NA	NA	NA	1.58	NA
	Sekine Y^9^	48	NA	87 ± 11	1.8 ± 0.3	NA	NA	65 ± 9	1.56	50 ± 10
Long‐ term	Korst RJ^7^	13	NA	NA	1.35	49	NA	NA	1.4	NA
	Korst RJ^7^	19	NA	NA	1.87	69	NA	NA	1.58	NA

FVC, forced vital capacity; FEV1, forced expiratory volume.

**Figure 1:**
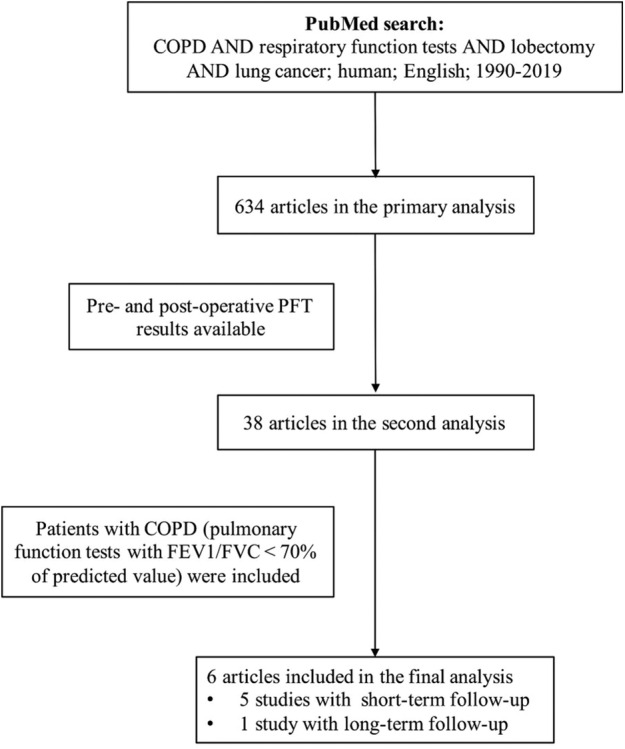
Diagram of literature selection.

A total of six studies, including 195 patients with COPD, provided values of lung function or changes of lung function before and after surgery (Fig 1). These patients met the following conditions:(i) Pulmonary function tests were performed before and after surgery (3–6 months for short‐term follow‐up, 1–2 years for long‐term follow‐up); (ii) The Eastern Cooperative Oncology Group performance status (PS) after surgery was 0 or 1; (iii) No patient received bilateral thoracic lobectomy. (iv) Patients had been diagnosed with COPD and early stage NSCLC. Due to the incomplete parameters of pulmonary function tests reported in previous studies, we focused on forced expiratory volume in the first second (FEV1) in our study.

## Results

**Figure 2:**
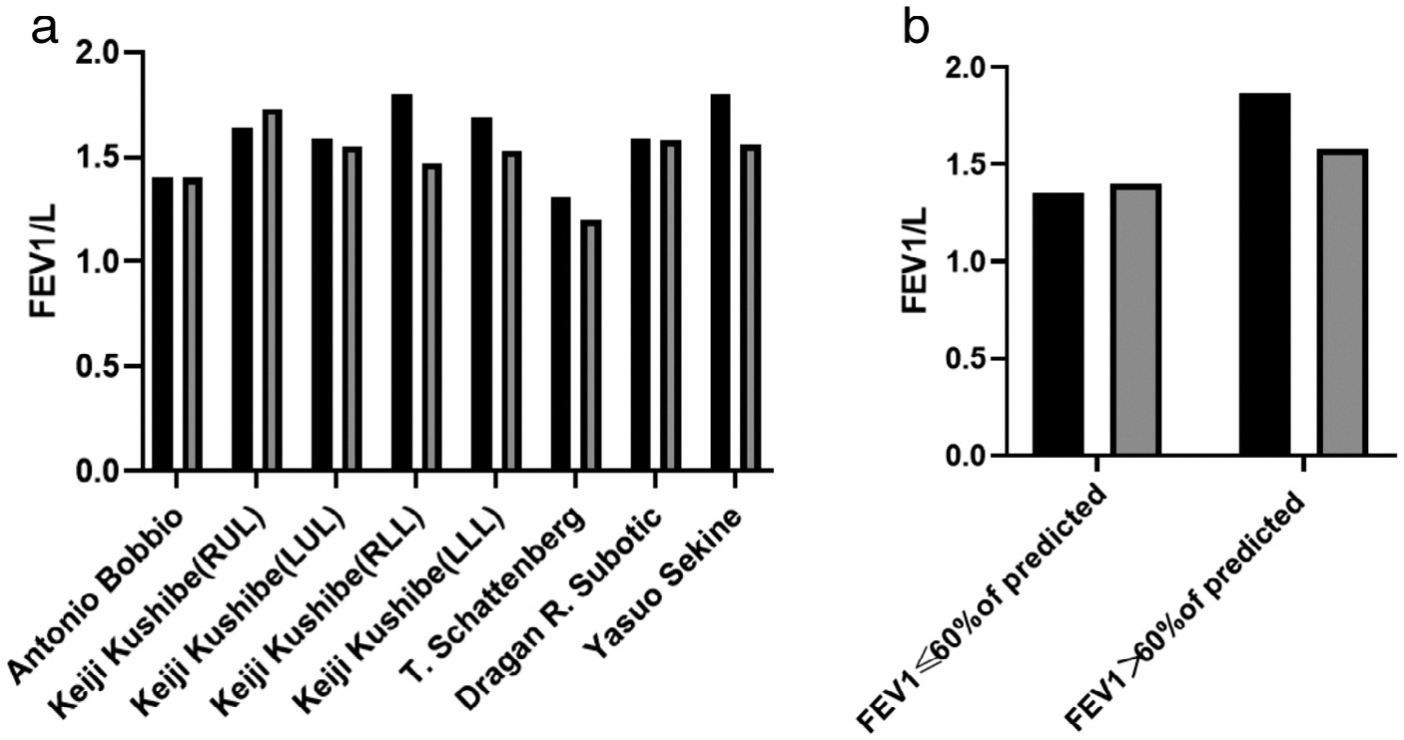
The changes of pulmonary function in lung cancer patients with COPD before and after operation. (**a**) The short‐term changes in FEV1. The study from Kushibe K and colleagues had four groups according to the location of lobectomy (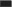 baseline value and 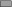 short‐term follow‐up). (**b**) The long‐term changes in FEV1. The study had two groups according to the difference value of preoperative FEV1 (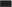 baseline value and 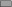 long‐term follow‐up).

Five out of six studies focused on the short‐term change of pulmonary function (within 3–6 six months) after lobectomy, and one study investigated the long‐term change in pulmonary function (within 1–2 years) after lobectomy. The pulmonary function of patients with COPD before and after operation within 3–6 months are shown in Table 2. The range of basic FEV1 was 1.31–1.80 L and the range of postoperative FEV1 was 1.20–1.73 L, and the average difference of FEV1 was −0.11 L (Fig 2a).

The pulmonary function of patients with COPD before and after operation within 1–2 years is reported in Table 2. The range of FEV1 of basic pulmonary function was 1.35–1.87 L and the postoperative range of FEV1 was 1.40–1.58 L. The average difference of FEV1 was −0.15 L (Fig 2b).

## Discussion

Impaired lung health, including COPD and lung cancer, represents a significant burden on global health. Lung cancer is the primary cause of death by malignancy in the world. COPD is the fourth leading cause of death globally, which is expected to become the third by 2020.^12^ An epidemiological survey by de Torres *et al*. discovered that the presence of COPD increases the risk of lung cancer by 4.5‐fold.^13^ Since most lung cancer patients are smokers and old age people, chronic lung diseases such as COPD often coexist and cause impaired lung function. Despite the significant progress which has been made in NSCLC treatment, surgery is still the first choice for early stage NSCLC patients. This study aimed to investigate the influence of lobectomy on the loss of pulmonary function in NSCLC patients with COPD.

We reviewed all the previous literature and found that there were a few relevant studies on this subject. We speculate that most NSCLC patients with COPD might receive nonsurgical treatments, such as chemotherapy and stereotactic body radiation therapy. According to previous studies, FEV1 decreases by 9%–17% after lobectomy for lung cancer patients with normal preoperative pulmonary function.^14^, ^15^ In our study, the average loss of FEV1 was 0.33 L (about 8.6%–19.0%) after lobectomy for the lung cancer patients without COPD (Table S1 and Fig S1). We also found that the postoperative change of FEV1 in patients with COPD was between −18.3% and 5%. suggesting that lobectomy does not further impair pulmonary function in patients with COPD. Because most patients in the Korst *et al*. study^7^ underwent open surgery, the FEV1 level decreased by 15.5% after 1–2 years. However, the choice of surgery for patients with COPD also needs to be carefully considered. Lobectomy may increase the incidence of postoperative complications or mortality for those patients with impaired preoperative pulmonary function.^16^, ^17^ It has been reported that patients with lung cancer and COPD are more likely to have postoperative complications than those without COPD, especially suffering long‐term air leaks.^18^, ^19^ Cardiovascular complications are also one of the serious postoperative complications for patients with COPD and the incidence rate is 3‐fold higher compared to those without COPD.^20^ Although there is a high incidence of postoperative complications in lung cancer patients with COPD, video‐assisted thoracoscopic surgery can significantly reduce the incidence of postoperative complications compared with traditional thoracotomy.^21^, ^22^ In addition, previous studies have shown that there is a good correlation between the predicted value and the measured value of pulmonary function after lobectomy.^23^, ^24^, ^25^ According to pulmonary function tests and radiographic assessments, patients with a pronounced emphysematous component of airway obstruction may have unchanged or even increased pulmonary function after lobectomy.^26^

The mechanism of pulmonary function change in patients with COPD seems to be different. A previous study shows that compared with those with normal pulmonary function, the loss of FEV1 is significantly reduced and the function of small airway is significantly improved in patients with COPD after pneumonectomy.^11^ First, there is no increase in FVC and FEV1 in patients with COPD. This may be due to the removal of functioning lung tissue. Second, upper lobectomy has better residual lung function than lower lobectomy. We speculated that, in COPD patients, upper lobectomy would have greater volume reduction effect than lower lobectomy, and would have little influence on the decrease in postoperative pulmonary function due to the anatomical features.^9^ After lobectomy in patients with COPD, the residual lung is overinflated and the diaphragm is elevated. Meanwhile, the mediastinum is displaced to the surgical side and the intercostal space is reduced to fill the space of the resected lung.^27^ As a result, the lung function of the patients is improved. Sekine *et al*.^9^ established a modified equation for predicting postoperative FEV1 (ppoFEV1 = 0.85 * preoperative FEV1 * [1‐S * 0.0526], where S = the number of bronchopulmonary segments removed). This new equation has the potential to expand the indication of standard surgery in patients with lung cancer and COPD. However, when assessing patients with impaired lung function, caution must be taken when evaluating patients with impaired lung function in the following two situations. On one hand, patients with a low preoperative FEV1 and COPD index (COPD index = percentage of preoperative FEV1 + measured FEV1/FVC) > 1.2 may have restrictive diseases and can be expected to sustain a 5% to 20% loss of function (FEV1) after lobectomy. On the other hand, patients with a COPD index of less than 1.0, where the relatively nonfunctioning lobe has not been resected seem to lose a large percentage of their FEV1 with resection of a functioning lobe.^7^

Pulmonary function exercise can significantly improve respiratory function, and has a protective effect on FEV1/FVC ratio, small airway function and blood oxygen saturation after operation.^28^ A good preoperative pulmonary rehabilitation is thought to contribute to significantly reduce dyspnea and improve exercise ability in patients with COPD, which may help to improve pulmonary function and allow radical surgery in these patients. As we know, the main postoperative pulmonary complications in patients with COPD are increased sputum secretion and impaired sputum excretion. Treatment with drugs such as salmeterol and tiotropium can reduce sputum secretion, whereas physical respiratory training can increase exercise endurance, both of which could improve pulmonary function postoperative management in these patients.

However, this study has several limitations. On one hand, there is a heterogeneity of the included studies which is mainly due to the difference of experimental design and the surgical procedure. On the other hand, most of the included studies are retrospective analyses which have a lower level of evidence (Fig S2).

In conclusion, with the development of minimally invasive thoracoscopic technology and enhanced recovery after surgery rapid rehabilitation, the extent of short‐term (3–6 months) loss of pulmonary function after operation is acceptable for lung cancer patients with COPD, which is comparable in the patients with normal pulmonary function. However, there may be a high risk of postoperative complications in NSCLC patients with COPD. Therefore, surgical treatment needs to be carefully considered for these patients.

## Disclosure

All authors have declared that there are no conflicts of interest.

## Supporting information


**Table S1.** Change of pulmonary function before and after lobectomy infor the patients without non‐COPD disease.

**Figure S1.** The short‐term changes of pulmonary function in lung cancer patients without COPD disease with non‐COPD before and after lobectomy operation. C. The short‐term changes in FEV1. The study from Kushibe K and colleagues has four groups according to the location of lobectomy.

**Figure S2.** Risk of bias analysis.
