# Supplementary material for: Outcomes of lobectomy on pulmonary function for early stage non‐small cell lung cancer (NSCLC) patients with chronic obstructive pulmonary disease (COPD)
Source: Thorac Cancer. 2020 May 6;11(7):1784–9. doi: 10.1111/1759-7714.13445 (PMC7592038; doi:10.1111/1759-7714.13445)
Supplement: Supplementary file 1 — Table S1. Change of pulmonary function before and after lobectomy infor the patients without non‐COPD disease. [file TCA-11-1784-s003.docx]

**Supplementary table 1. The short-term changes of pulmonary function in lung cancer patients without COPD disease before and after lobectomy**

|  | **First Author** | **Number** | **Baseline Value** | | | | **Postoperative value** | | | | |
| --- | --- | --- | --- | --- | --- | --- | --- | --- | --- | --- | --- |
|  |  |  | **FVC** | **FVC%** | **FEV1** | **FEV1%** | **FVC** | **FVC%** | **FEV1** | | **FEV1%** |
| Short term | Kushibe K^[27]^ | 35 | 3.22 ±0.85 | 104.2 ±17.6 | 2.54 ±0.78 | 104.1 ±20.1 | 2.96 | NA | 2.22 | NA | |
|  | Kushibe K^[27]^ | 35 | 1.91 ±0.76 | 99.8 ±13.7 | 2.34 ±0.64 | 109.2 ±14.3 | 2.40 | NA | 1.89 | NA | |
|  | Kushibe K^[27]^ | 34 | 3.18 ±0.92 | 100.6 ±19.3 | 2.51 ±0.74 | 103.5 ±24.3 | 2.61 | NA | 2.11 | NA | |
|  | Kushibe K^[27]^ | 29 | 3.01 ±0.58 | 99.8 ±13.7 | 2.39 ±0.49 | 103.4 ±24.7 | 2.61 | NA | 2.08 | NA | |
|  | Subotic DR^[22]^ | 47 | NA | NA | 2.852 ±4.79 | NA | NA | NA | 2.65 | NA | |
