# Supplementary figures and images for: Outcomes of lobectomy on pulmonary function for early stage non‐small cell lung cancer (NSCLC) patients with chronic obstructive pulmonary disease (COPD)
Source: Thorac Cancer. 2020 May 6;11(7):1784–9. doi: 10.1111/1759-7714.13445 (PMC7592038; doi:10.1111/1759-7714.13445)

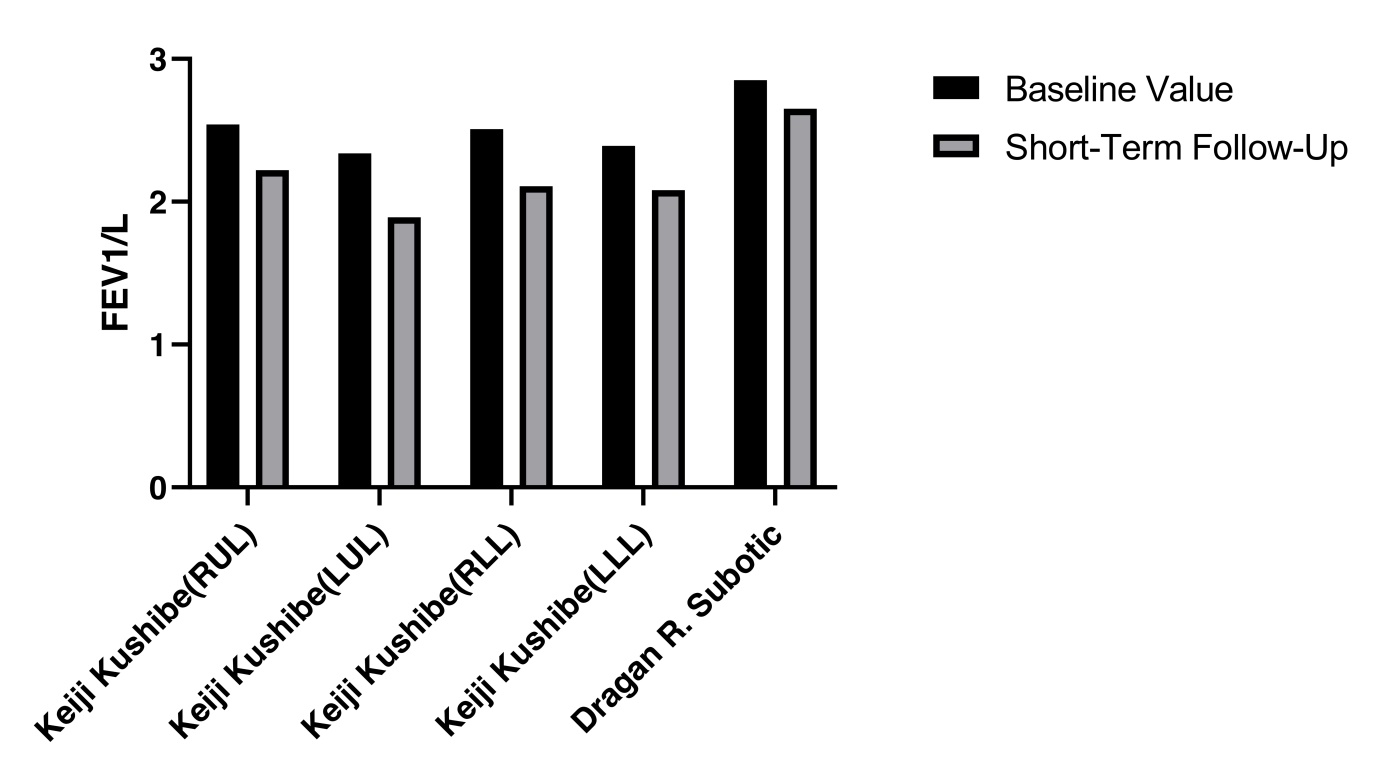

Supplement: Supplementary file 2 — Figure S1. The short‐term changes of pulmonary function in lung cancer patients without COPD disease with non‐COPD before and after lobectomy operation. C. The short‐term changes in FEV1. The study from Kushibe K and colleagues has four groups according to the location of lobectomy. [file TCA-11-1784-s001.docx]

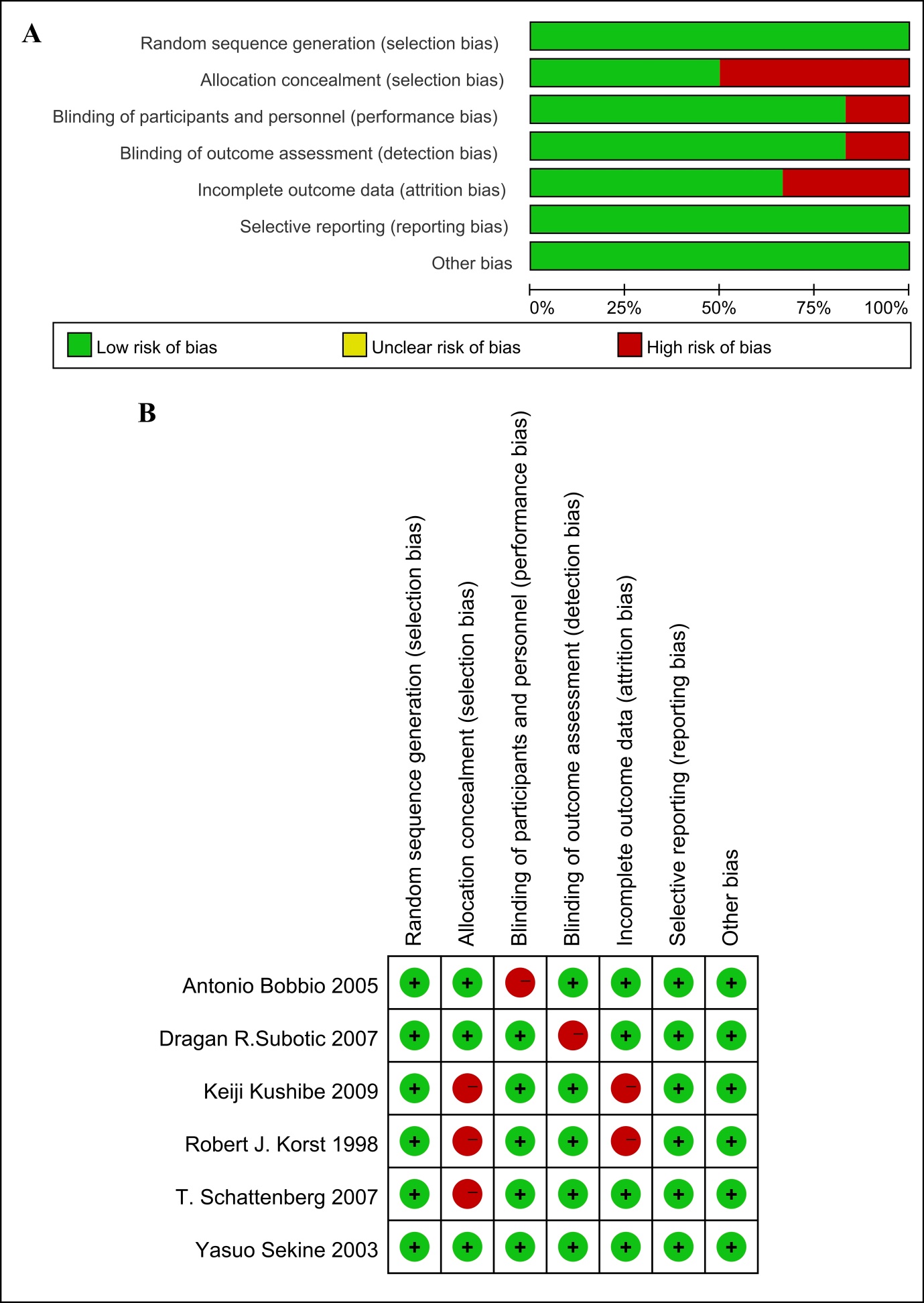

Supplement: Supplementary file 3 — Figure S2. Risk of bias analysis. [file TCA-11-1784-s002.docx]
